# Assessment of ganglion cell complex thickness and its correlation with retinal sensitivity using microperimetry 6 months after epiretinal membrane surgery

**DOI:** 10.1186/s40942-024-00576-y

**Published:** 2024-08-23

**Authors:** Leonardo Provetti Cunha, Aline Mota Freitas Matos, Raphael Lucas Sampaio Defina, Luciana Virgínia Ferreira Costa-Cunha, Leandro Cabral Zacharias, Rony Carlos Preti, Mário Luiz Ribeiro Monteiro

**Affiliations:** 1 Division of Ophthalmology, Federal University of Juiz de Fora Medical School, Avenida Barão Do Rio Branco, 4051. Bom Pastor, Juiz de For a, Minas Gerais, 36021-630 Brazil; 2 Juiz de Fora Eye Hospital, Minas Gerais, Brazil; 3 Division of Ophthalmology and the Laboratory of Investigation in Ophthalmology (LIM 33), University of São Paulo Medical School, São Paulo, Brazil

**Keywords:** Epiretinal membrane, Macula, Ganglion cell complex, Ganglion cell/inner plexiform later, Microperimeter/microperimetry, Retinal sensitivity, Optical coherence tomography, Pars-plana posterior vitrectomy, Retina

## Abstract

**Purpose:**

To verify the correlation between the full-macular and the ganglion cell complex (GCC) thickness measurements and retinal sensitivity (RS) assessed by microperimetry (MP) 6 months after surgical peeling for idiopathic epiretinal membrane (ERM).

**Methods:**

Forty-three were submitted to pars-plana posterior vitrectomy (PPV) with concomitant peeling of internal limiting membrane (ILM) for idiopathic ERM treatment. Best-corrected visual acuity (BCVA) and 3D volumetric high-definition optical coherence tomography (OCT) imaging were preoperatively acquired. Six months after the surgery, BCVA, OCT imaging, and RS measured by MP were assessed. For the OCT parameters, we analyzed both the full-macular and the ganglion cell layer complex (GCC) thicknesses. The MP parameters tested were 44 points covering 20 central degrees (6 mm), with direct correspondence with the nine sectors of the OCT-ETDRS map. This approach enables the direct topographic correlation between the structure and functional measurements. The OCT and MP exam measurements were also performed in 43 eyes of age-matched healthy controls. Correlations between BCVA, RS, and OCT parameters were examined.

**Results:**

All patients exhibited a substantial improvement in visual acuity following surgery. The RS parameters were significantly lower in patients compared to the controls. The full-macular thickness measurements were thicker than controls preoperatively and significantly reduced postoperatively; however, remaining significantly higher than controls, in the 4 inner sectors, at the fovea and for the average macular thickness. Preoperative GCC measurements were higher than those in controls. There was a significant reduction in GCC thickness in all sectors postoperatively, especially in the outer sectors, as well as in the average macular thickness. A positive correlation was found between full-macular and GCC thickness and RS postoperatively in several sectors.

**Conclusions:**

Our results demonstrate that ERM peeling can improve visual acuity in the postoperative period. However, RS may not fully restore, remaining significantly lower when compared to the controls. Both full-macular and GCC thickness measurements were reduced 6 months after surgery. However, significant thinning of the GCC thickness was observed when compared to the normal control eyes, indicating the occurrence of some degree of ganglion cell layer atrophy. We have demonstrated significant correlations among various OCT thickness parameters, particularly for GCC measurements. We believe that GCC integrity may play an important role in visual function after ERM surgery, and that MP may help better understand the correlations between structural and functional findings following ERM surgery.

## Background

The idiopathic epiretinal membrane (ERM) manifests as the development of fibrocellular tissue on the inner surface of the retina, typically associated with the proliferation and migration of glial cells. These cellular events may result in tractional forces being applied to the underlying retina, consequently causing distortion, thickening, and functional impairment of the macula [1].

Pars plana vitrectomy (PPV) and membrane peeling surgery represents the standard surgical approach, aiming to relieve traction on the macula and improve visual function [2–4]. Postoperative visual outcomes may exhibit considerable variability, ranging from mild improvement to significant restoration of visual acuity (VA). However, metamorphosis and visual complaints may persist after surgery, possibly related to remaining retinal structural changes [2, 5].

Spectral-domain optical coherence tomography (OCT) serves as the primary diagnostic modality for evaluating ultrastructural alterations in patients with ERM during both preoperative and postoperative phases. Its utility extends to estimating disease severity, predicting visual recovery potential, and assessing retinal status following surgical intervention. Numerous previous studies have consistently shown a correlation between full-macular thickness measurements and the severity of the disease, as well as the extent of visual impairment post-surgery [6–8].

While ERM can indeed impair all retinal layers, its origin from the retinal surface often predominantly impacts the inner retina. Therefore, directing investigative efforts toward the inner retina, i.e., ganglion cell/inner plexiform layers, could yield more enlightening results. While certain prior studies have explored the relationship between inner retinal thickness measurements and postoperative visual function in ERM patients, most investigations have assessed this correlation using VA or standard automated perimetry mean sensitivity measurements [8–11].

In a recently published study, we investigated the correlation between retinal sensitivity (RS) measured by microperimetry (MP) and OCT parameters in eyes that underwent ERM peeling [12]. Our findings revealed a significant decrease in RS values following surgery. In this study, certain OCT biomarkers, such as the presence of disorganization of retinal inner layers (DRIL) both pre-and post-surgery, as well as the occurrence of microcysts and outer retinal changes post-surgery, were associated with poorer visual outcomes. Notably, we employed an innovative approach for correlational analysis, utilizing direct topographic comparisons between MP and OCT parameters. Specifically, RS analyses were conducted and correlated within each of the 9 sectors defined by the OCT ETDRS map. However, despite demonstrating the impact of these biomarkers on visual function, we believe that other variables can influence visual recovery after ERM peeling surgery, such as the degree of impairment of the inner retinal layers both preoperatively and postoperatively.

So, the present study aims to verify the correlation between the thickness of the ganglion cell complex (GCC) and retinal sensitivity (RS) assessed by MP 6 months after surgical peeling for idiopathic ERM.

## Methods

### Study design

An observational prospective study enrolled patients undergoing PPV to remove the ERM with the simultaneous internal limiting membrane (ILM) peeling. Approval for the study was obtained from the Research Ethics Committee of the Federal University of Juiz de Fora (CAAE number 12296919.0.0000.5147), and informed consent was obtained from all participants.

Patients with ERM and visual loss or metamorphopsia were included in the study. ERM diagnosis relied on fundus and optical coherence tomography (OCT) images. Patients who were previously pseudophakic or underwent cataract extraction with an intraocular lens (IOL) implantation concurrently with vitrectomy surgery were selected. Additional inclusion criteria comprised ages ranging between 40 and 85 years, refractive errors between 5 spherical diopters and 3 cylindrical diopters, preoperative best-corrected visual acuity (BCVA) ranging from 20/25 to 20/200, preoperative intraocular pressure less than or equal to 21 mmHg, and good cooperation for OCT examination. Age-matched healthy control eyes were selected for comparison of macular thickness parameters before and after surgery, as well as RS results 6 months post-surgery.

Exclusion criteria included non-idiopathic ERM, intra or postoperative complications, prior history of rhegmatogenous retinal detachment, posterior vitrectomy, trabeculectomy, or complicated cataract surgery, previous intravitreal injections, corneal opacity, glaucoma, other optic disc neuropathies, diabetic retinopathy, arterial or venous occlusions, or any macular diseases besides ERM. Additionally, individuals with an axial diameter greater than 25 mm or systemic diseases, excluding well-controlled systemic arterial hypertension, were excluded from the study.

### Ocular examination

All patients underwent a comprehensive ophthalmological examination before surgery and at months 1, 3, and 6 postoperatively. The eye examination included an assessment of BCVA, intraocular pressure measured with Goldmann applanation tonometry, slit lamp biomicroscopy, and fundus biomicroscopy. BCVA measurements were evaluated using a Snellen chart and subsequently converted to a logarithm of the minimum angle of resolution units (logMAR) for statistical analyses.

### Intraoperative procedures

In all patients enrolled, a 25-gauge PPV was performed using a 7500 cpm vitrectomy probe (Constellation Vision System, Alcon), either as a standalone procedure or in conjunction with cataract extraction and implantation of a foldable single-piece intraocular lens (IOL). Following removal of the vitreous body and posterior hyaloid, simultaneous staining of the ERM and ILM was achieved using MembraneBlue Dual (D.O.R.C. Dutch Ophthalmic Research Center, Zuidland, Netherlands), chosen for its ability to stain both structures simultaneously.

Subsequently, the ERM was meticulously grasped and peeled utilizing **GRIESHABER REVOLUTION DSP® ILM forceps**(Alcon Surgical, Fort Worth, TX), followed by ILM removal akin to a capsulorhexis procedure. A second round of staining post-ILM removal was conducted at the surgeon's discretion to ensure complete removal. The extent of ILM peeling was ensured to cover the entire macular area. Following a thorough review of the vitreous base and retinal periphery, fluid-air exchange was performed. All surgical procedures were performed by the same surgeon (L.P.C) at Juiz de Fora Eye Hospital.

### Optical coherence tomography

The patients underwent OCT examination before and at 1, 3, and 6 months postoperatively. Using the device's internal fixation under mydriasis, SS-OCT high-resolution B-scan sectional images (up to 4096 pixels) and 3D volumetric images covering up to 7 × 7 mm of the macular area with a scan density of 512 × 256 were acquired.

**Fig. 1:**
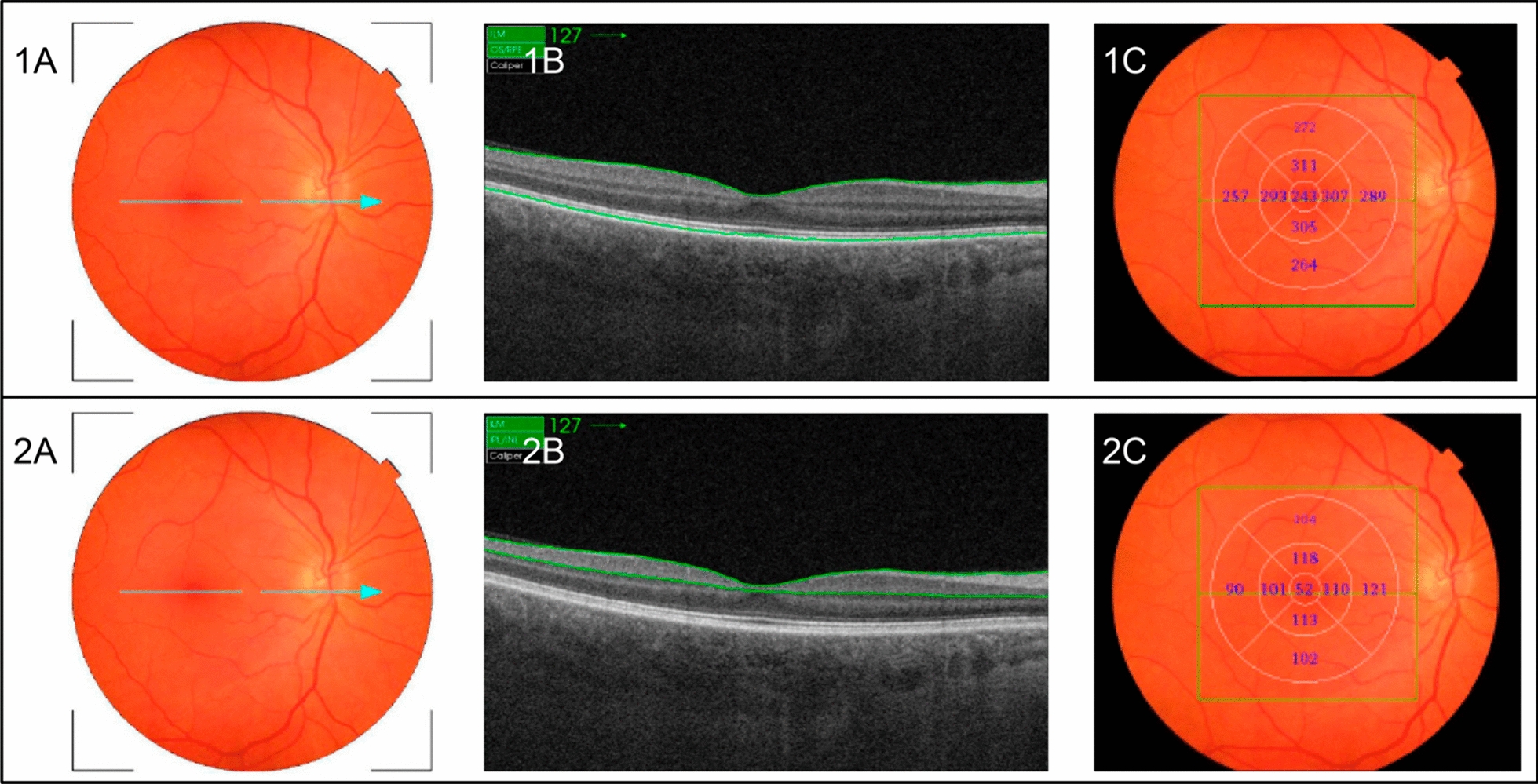
Representative images of a control individual. **A**: Fundus image displaying the macular region, with a blue arrow denoting the OCT-scanned area through the macular center. **B**: Cross-sectional OCT image illustrating full-macular thickness between the ILM and the transition from the neurosensory retina to the retinal pigment epithelium (green lines). **C**: Fundus image of the same individual with OCT full-macular thickness measurements, segmented into nine sectors according to the ETDRS map. 2A: Fundus image showing the macular area, with a blue arrow indicating the OCT-scanned area through the macular center. 2B: Cross-sectional OCT image demonstrating ganglion cell complex (GCC) thickness between the ILM and the lower boundary of the ganglion cell/internal plexiform layer (green lines). 3C: Fundus image of the same individual with GCC thickness measurements, divided into nine sectors based on the ETDRS map

Automatically determined by the device, the full-macular thickness lies between the ILM and the transition between the neurosensory retina and the retinal pigment epithelium (RPE) (green lines) (Fig. 1). The GCC was also automatically identified by the built-in software, positioned between the ILM and the lower boundary of the ganglion cell/internal plexiform layer (GCL/IPL). This complex comprises macular retinal nerve fiber layers (RNFL) plus GCC/IPL (Figs. 1 e 2).

The ERM OCT findings were graded into four stages, as previously described [12]. According to this classification, in stage 1, the foveal depression is present, and the retinal layers are well-defined. In stage 2, the foveal depression is absent, but the retinal layers remain well-defined. In stage 3, the foveal pit is absent, yet all retinal layers are clearly identified. In stage 4, the foveal pit is absent, and disorganization of the retinal inner layers (DRIL) is present. Patients with stage 1 ERM were not included in the study.

**Fig. 2:**
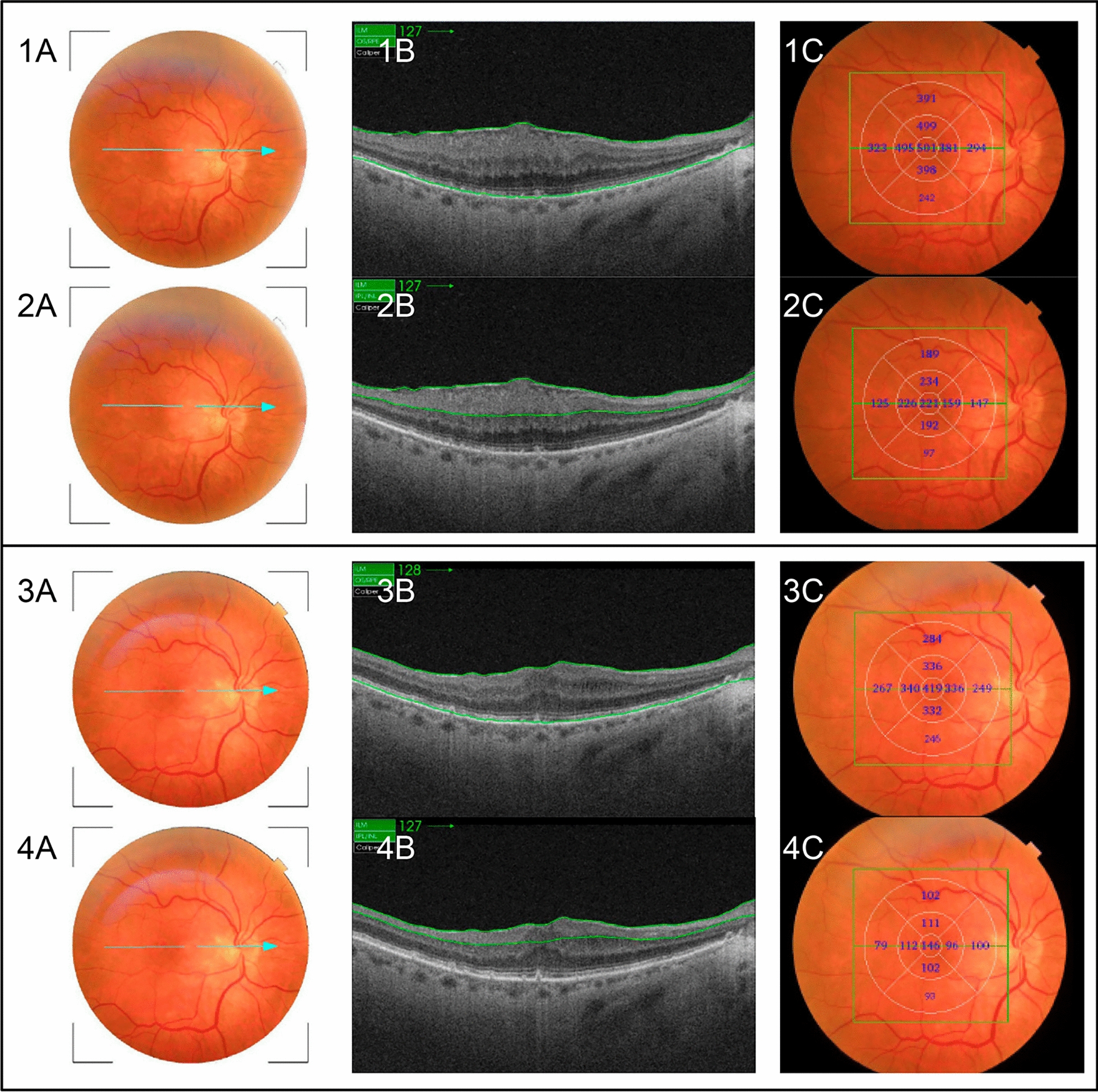
Representative images of a patient with an idiopathic epiretinal membrane (ERM) before surgery, showing full-macular thickness measurements. 2: Images of the same patient as in image 1, with an idiopathic ERM before surgery, showing GCC thickness measurements. 3: Images of the same patient 6 months after epiretinal membrane surgery, showing total macular thickness measurements. 4: Images of the same patient 6 months after epiretinal membrane surgery, showing GCC thickness measurements. **A**: Fundus image showing the macular area. The blue arrow represents the OCT-scanned area through the center of the macula. **B**: Cross-sectional OCT images. **C**: Fundus image with OCT thickness measurements according to the nine sectors of the ETDRS map

The examiner evaluated the images based on their objective and subjective quality, with no abrupt eye movements causing artifacts or dark lines due to blinking. All images underwent scrutiny for artifacts generated during acquisition or segmentation errors, with any affected images being discarded and replaced with new acquisitions. Special emphasis was placed on the segmentation of the OCT B-scan images. Any images exhibiting segmentation errors or notable signs of DRIL in the postoperative OCT images were excluded. This exclusion was considered necessary as it hindered the accurate identification and segmentation of the GCC layers. The device's software automatically computed parameters for measuring the full-macular thickness. The analysis of total retinal thickness measurements in µm followed division into nine sectors of the ETDRS map (Figs. 1 and 2).

### Microperimetry test

**Fig. 3:**
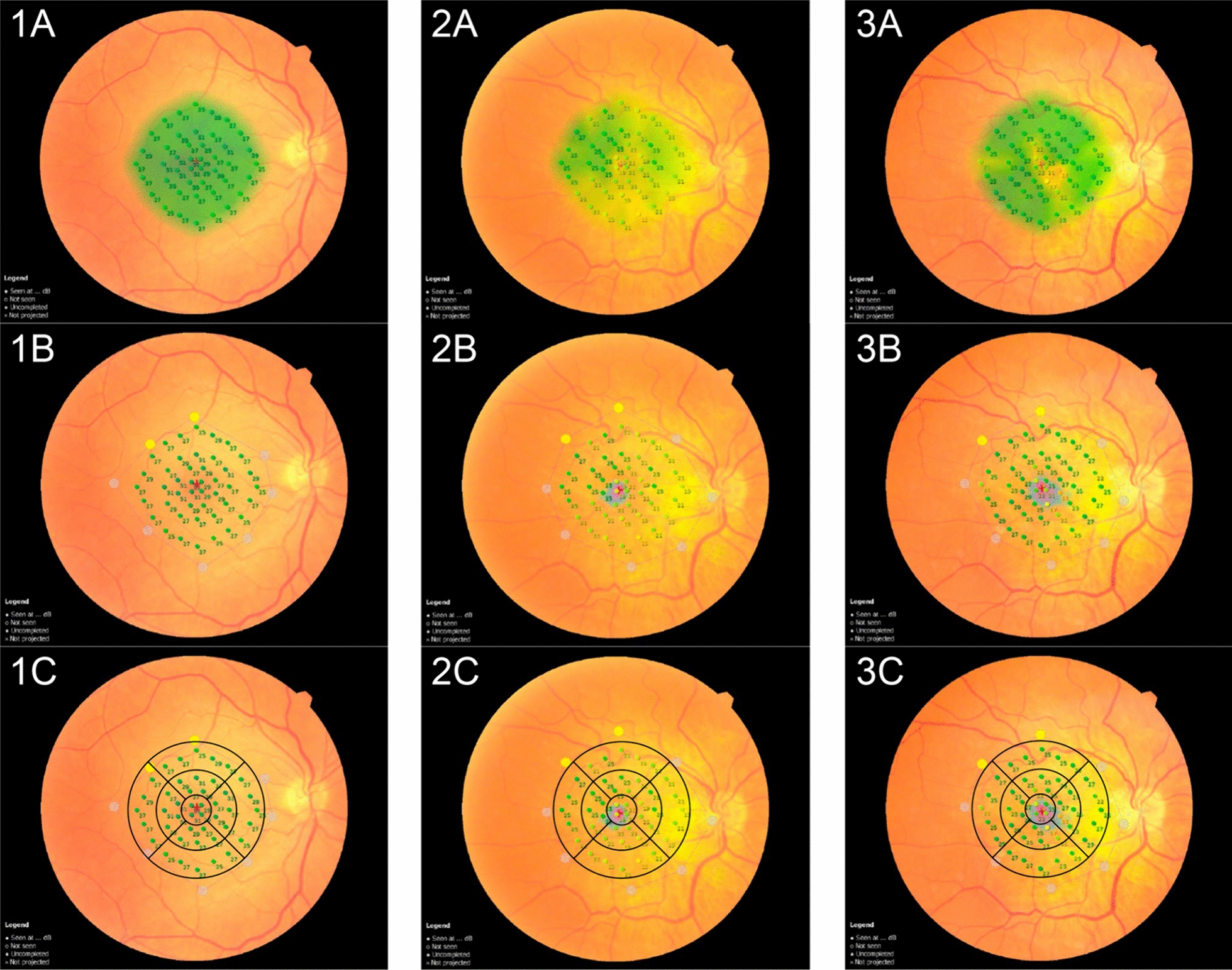
Representative images of the microperimetry (MP) test. The exam combines a fundus camera image with an overlaid microperimetry grid, featuring 44 tested points covering 20 central degrees (10 degrees from the center of the fovea in each direction), encompassing a 6 mm diameter in the macular area. 1: Representation of MP in a normal control eye. 2: Example of an MP test in a patient with an idiopathic ERM before surgery. 3: The same patient as in 2, 6 months after ERM surgery. **A**: The green color represents retinal sensitivity (RS) responses within normal limits. Yellow indicates points of borderline retinal sensitivity, and red indicates areas outside normal limits (not represented in these images). **B**: Projection of the points tested by MP in direct correspondence with the fundus image. **C**: OCT ETDRS map with a direct topographic projection overlaid on the MP-tested area. Each inner and outer ETDRS map sector’s thickness measurements, as measured by OCT, contain five RS-tested points, while the central circle (1 mm) contains four RS-tested points

The RS was evaluated using MP testing in all patients 6 months post-surgery. The MP parameters encompassed 44 points distributed across a 20-degree central field, covering a 6 mm diameter macular region that directly corresponds to the nine sectors of the ETDRS map (Fig. 3), as previously published [12]. This arrangement enabled direct topographic correlation between RS retinal sensitivity measured by MP and the thickness measurements (in µm) of the full-macular and GCC across the nine sectors of the ETDRS map.

Each inner and outer sector of the ETDRS map analyzed by OCT contained 5 points tested for retinal sensitivity, while the central circle (1 mm) had 4 tested points (Fig. 3). The MP stimulus employed was Goldmann size III, projected for 200 ms, against a white-back white background with a luminance of 1.27 CD/m [2] (4 Apostilb ASB). The MP's maximum luminance reached 10,000 ASB, with stimulus attenuation programmed between 0 in decibels (dB), representing maximum luminance, and 34 dB, minimal stimulus luminance. A 4–2 threshold strategy (Full-Threshold Staircase) was utilized. The MP test incorporated an eye-tracking system to compensate for eye movements and monitor fixation. All examinations were conducted following mydriasis.

The device's software automatically computed the mean average of all 44 threshold points measured in dB for each patient, representing the mean retinal sensitivity in dB. Mean RS responses were further calculated for each of the nine sectors of the ETDRS map, as previously described [12]. Areas unable to detect the maximum visual stimulus threshold were classified as absolute scotomas (0 dB). A 4-degree red cross served as a fixation target for the exam. To minimize the effect of the learning curve, we initially performed the microperimetry exam on the non-operated contralateral eye.

### Statistical analysis

The McNemar test was employed to compare the proportions of categorical variables before and after surgery. The Chi-Square test was utilized to compare proportions between patient and control groups. Normality and equality of variances were assessed using the Kolmogorov–Smirnov test and the Levene test, respectively. Pearson's correlation coefficient was calculated to evaluate relationships between continuous variables. Cohen's Kappa coefficient of agreement determined inter-observer agreement for qualitative variables and ERM classification [12].

The independent samples t-test was conducted to compare MP and OCT parameters between patients and controls, while the paired t-test was utilized to compare differences in parameters before and after surgery. Analysis of variance (ANOVA) was performed to test differences in BCVA according to ERM presence, with the Bonferroni post hoc test for multiple comparisons. Receiver operating characteristic (ROC) curve analysis was used to assess the ability of OCT parameters to discriminate patients from controls. All analyses were conducted using IBM SPSS Statistics (version 22.0; IBM Corporation).

## Results

A total of 43 patients, aged between 52 and 85 years (mean age: 69.4 ± 4.4 years), met the inclusion criteria and were followed for an average duration of 9.6 ± 6.6 months after surgery. Additionally, 43 age- and sex-matched healthy individuals were selected as controls for the study. Table 1 outlines the clinical characteristics of both patients and controls. No statistically significant differences were observed between the groups in terms of gender, age, or intraocular pressure. However, there was a statistically significant difference in the BCVA between patients and controls in pre and postoperative period.

**Table 1:** Clinical characteristics of patients and controls

Variables	Category/measure	Patients (n = 43)	Controls (n = 43)	p-value
Age (years)	–	69.4 ± 4.4	68.3 ± 7.9	0.45^a^
Gender	Women	24 (55.8%)	24(55.8%)	1.00^b^
	Men	19 (44.2%)	19 (44.2%)	
Visual Acuity (LogMAR)	Pre-op	0.37 ± 0.19	0.01 ± 0.04	** < 0.001**^a^
	Post-op	0.04 ± 0.09		**0.03**^a^
		< 0.001^c^		
Intraocular Pressure		14.6 ± 3.5	13.6 ± 2.4	0.12^a^
Time after surgery (months)	–	9.6 ± 6.6		
ERM classification				
1	1	0 (0.0%)		
2	2	12 (27.9%)		
3	3	21 (48.8%)		
4	4	10 (23.3%)		
Phakic status				
Phakic eyes	4			
Pseudophakic	17			
PPV + facectomy	22			

Bold values denote statistical significance at the p < 0.05. ERM epiretinal Membrane. ^a^Independent t-test, ^b^Chi-square test, ^c^McNemar test, ^d^Paired t-test (BCVA pre versus postoperative)

Table 2 illustrates the full-macular thickness measurements in patients compared to the controls. Before surgery, all patients exhibited significantly higher full-macular thickness measurements compared to the controls. After surgery, there was a notable reduction in all nine OCT ETDRS map sectors, as well as in the average macular thickness and macular volume (p < 0.001). Despite this reduction, postoperative macular thickness measurements remained elevated compared to controls in the four inner sectors, the fovea, and the average macular thickness and macular volume. Conversely, there was no significant difference observed in the four outer ETDRS map sectors six-months after surgery.

**Table 2:** Mean pre- and postoperative full-macular thickness measurements (in µm) obtained by OCT in patients and controls, divided into nine sectors, along with average thickness and macular volume, with corresponding values of areas under the ROC curve

OCT full-macular thickness (µm)	Patients (n = 43)	Controls (n = 43)	p-value	AUC
Fovea				
Pre-op	455.4 ± 67.2		< 0.001*	0.99 (0.98 – 1.00)
Post-op	373.5 ± 58.7	241.1 ± 34.3	< 0.001*	0.96 (0.91 – 1.00)
p-value	< 0.001*			
Temporal Inner				
Pre-op	419.9 ± 58.1		< 0.001*	0.99 (0.97 – 1.00)
Post-op	328.9 ± 56.8	298.1 ± 16.1	0.001*	0.73 (0.62 – 0.84)
p-value	< 0.001*			
Superior inner				
Pre-op	427.9 ± 54.1		< 0.001*	0.99 (0.98 – 1.00)
Post-op	344.5 ± 35.1	309.8 ± 17.1	< 0.001*	0.83 (0.75 – 0.92)
p-value	< 0.001*			
Nasal Inner				
Pre-op	410.7 ± 52.8		< 0.001*	0.94 (0.89 – 1.00)
Post-op	358.5 ± 36.2	310.6 ± 17.9	< 0.001*	0.89 (0.81 – 0.96)
p-value	< 0.001*			
Inferior inner				
Pre-op	397.1 ± 55.3		< 0.001*	0.93 (0.88 – 0.99)
Post-op	334.7 ± 31.5	308.2 ± 20.5	< 0.001*	0.76 (0.66 – 0.86)
p-value	< 0.001*			
Temporal outer				
Pre-op	312.5 ± 51.8		< 0.001*	0.90 (0.83 – 0.98)
Post-op	259.2 ± 27.1	254.4 ± 11.5	0.30	0.55 (0.43 – 0.68)
p-value	< 0.001*			
Superior outer				
Pre-op	327.0 ± 42.7		< 0.001*	0.91 (0.84 – 0.98)
Post-op	279.0 ± 29.4	269.4 ± 14.6	0.06	0.64 (0.52 – 0.76)
p-value	< 0.001*			
Nasal outer				
Pre-op	328.4 ± 39.6		< 0.001*	0.88 (0.80 – 0.95)
Post-op	286.9 ± 26.9	284.5 ± 15.2	0.61	0.53 (0.41 – 0.66)
p-value	< 0.001*			
Inferior outer				
Pre-op	297.9 ± 45.5		< 0.001*	0.76 (0.65 – 0.88)
Post-op	258.7 ± 24.7	259.8 ± 13.5	0.80	0.50 (0.38 – 0.63)
p-value	< 0.001*			
Average thickness				
Pre-op	342.8 ± 33.6		< 0.001*	0.97 (0.92 – 1.00)
Post-op	289.1 ± 23.1	275.1 ± 13.3	0.001*	0.71 (0.60 – 0.82)
p-value	< 0.001*			
Macular volume				
Pre-op	9.7 ± 0.9		< 0.001*	0.97 (0.92 – 1.00)
Post-op	8.2 ± 0.6	7.8 ± 0.4	0.001*	0.71 (0.60 – 0.82)
p-value	< 0.001*			

OCT: Optical coherence tomography; AUC: area under the ROC (receiver operating characteristic) curve; *: represents p < 0.05. by paired Student's t test (pre- versus post-surgery) and for independent samples (patients versus controls). pre-op: preoperative. post-op: postoperative

Table 3 displays the GCC thickness measurements in patients and controls. Preoperative GCC thickness was significantly higher than in controls. Following surgery, GCC thickness reduced across all 9 ETDRS map sectors, as well as for the overall average thickness (p < 0.001). Postoperatively, GCC thickness remained significantly higher compared to controls only in the foveal region. Conversely, GCC thickness was significantly reduced in all four outer sectors (temporal, superior, nasal, and inferior).

**Table 3:** Mean pre- and postoperative GCC thickness measurements (in µm) obtained by OCT in patients and controls, divided into nine sectors, along with average thickness and macular volume, with corresponding values of areas under the ROC curve

GCC thickness (µm)	Patients (n = 43)	Controls (n = 43)	p-value	AUC
Fovea				
Pre-op	189.6 ± 54.1		< 0.001*	0.99 (0.99 – 1.00)
Post-op	109.1 ± 36.9	45.8 ± 12.9	< 0.001*	0.98 (0.95 – 1.00)
p-value	< 0.001*			
Temporal inner				
Pre-op	184.5 ± 40.3		< 0.001*	0.99 (0.98 – 1.00)
Post-op	103.5 ± 27.4	106.9 ± 6.7	0.44	0.60 (0.48 – 0.73)
p-value	< 0.001*			
Superior inner				
Pre-op	193.8 ± 33.0		< 0.001*	0.99 (0.99 – 1.00)
Post-op	116.9 ± 28.7	118.0 ± 7.4	0.81	0.57 (0.44 – 0.70)
p-value	< 0.001*			
Nasal inner				
Pre-op	182.5 ± 34.7		< 0.001*	0.96 (0.92 – 1.00)
Post-op	120.0 ± 25.6	113.2 ± 8.0	0.11	0.57 (0.44 – 0.70)
p-value	< 0.001*			
Inferior Inner				
Pre-op	180.2 ± 39.8		< 0.001*	0.91 (0.84 – 0.99)
Post-op	112.5 ± 18.4	118.0 ± 10.6	0.09	0.62 (0.49 – 0.74)
p-value	< 0.001*			
Temporal outer				
Pre-op	122.6 ± 29.7		< 0.001*	0.86 (0.78 – 0.94)
Post-op	78.6 ± 16.0	91.7 ± 8.4	< 0.001*	0.84 (0.75 – 0.92)
p-value	< 0.001*			
Superior outer				
Pre-op	138.3 ± 27.0		< 0.001*	0.87 (0.79 – 0.96)
Post-op	94.7 ± 19.1	103.1 ± 8.9	0.01*	0.72 (0.61 – 0.83)
p-value	< 0.001*			
Nasal outer				
Pre-op	152.5 ± 26.5		< 0.001*	0.91 (0.85 – 0.97)
Post-op	110.4 ± 19.3	117.9 ± 10.2	0.03*	0.62 (0.49 – 0.74)
p-value	< 0.001*			
Inferior outer				
Pre-op	129.9 ± 37.2		< 0.001*	0.78 (0.67 – 0.89)
Post-op	90.6 ± 15.9	103.5 ± 10.9	< 0.001*	0.78 (0.68 – 0.88)
p-value	< 0.001*			
Average thickness				
Pre-op	139.1 ± 17.9		< 0.001*	0.96 (0.92 – 1.00)
Post-op	96.0 ± 14.7	104.3 ± 7.8	0.002*	0.71 (0.61 – 0.82)
p-value	< 0.001*			

GCC: ganglion cell complex (which corresponds to the macular retinal nerve fiber layer (RNFL) plus ganglion cell layer/inner plexiform); AUC: area under the ROC (receiver operating characteristic) curve; *: represents p < 0.05. by paired Student's t test (pre- versus post-surgery) and for independent samples (patients versus controls). pre-op: preoperative. post-op: postoperative

In terms of MP findings post-surgery, a statistically significant difference in RS was observed between patients and controls across all sectors (see Table 4). Additionally, following surgery, RS was significantly lower in patients across all sectors and for the mean RS.

**Table 4:** . The mean values of retinal sensitivity measured in decibels (dB) obtained through postoperative MP, divided into 9 sectors along with the mean sensitivity. Additionally, it provides the corresponding values of the areas under the ROC curve

Retinal sensitivity (dB)	Patients (n = 43)	Controls (n = 43)	p-value	AUC
Fovea	22.3 ± 5.3	27.1 ± 2.8	< 0.001*	0.81 (0.71 – 0.91)
Temporal inner	24.4 ± 4.2	27.6 ± 1.5	< 0.001*	0.78 (0.66 – 0.89)
Superior inner	23.9 ± 4.6	26.8 ± 1.8	< 0.001*	0.73 (0.61 – 0.85)
Nasal inner	23.4 ± 5.1	26.9 ± 1.9	< 0.001*	0.74 (0.62 – 0.86)
Inferior inner	23.1 ± 5.2	26.5 ± 2.0	< 0.001*	0.73 (0.61 – 0.85)
Temporal outer	21.2 ± 7.1	26.7 ± 1.8	< 0.001*	0.78 (0.66 – 0.89)
Superior outer	22.3 ± 5.6	25.9 ± 2.2	< 0.001*	0.76 (0.64 – 0.87)
Nasal outer	22.9 ± 6.2	26.0 ± 2.1	0.005*	0.67 (0.54 – 0.80)
Inferior outer	21.9 ± 6.1	25.4 ± 2.3	0.001*	0.66 (0.53 – 0.80)
Mean sensitivity	22.7 ± 5.1	26.5 ± 1.8	< 0.001*	0.78 (0.68 – 0.89)

dB: decibel, AUC area under the ROC (receiver operating characteristic) curve. *: represents p < 0.05 and represents unpaired Student's t-test for independent samples (patients versus controls)

There was a positive and statistically significant correlation between pre- and post-surgery visual acuity (r = 0.50; p = 0.005). In other words, the worse the preoperative visual acuity, the worse the visual acuity will be after surgery.

No correlation was found between the ERM classification and average foveal sensitivity (p = 0.33) or average global sensitivity (p = 0.56).Our results demonstrate a positive correlation between preoperative VA (logMAR) and postoperative foveal thickness (Table 5). Specifically, worse preoperative VA is associated with greater postoperative foveal thickness. Additionally, a negative correlation was observed between preoperative VA and GCC average thickness after surgery (Table 5), meaning that worse preoperative VA corresponds to thinner GCC thickness postoperatively. Furthermore, there was a negative correlation between preoperative VA and mean foveal sensitivity in the postoperative period, indicating that worse preoperative VA leads to lower retinal sensitivity in the fovea after surgery (Table 5).

**Table 5:** Correlation between OCT full-macular and GCC thickness (pre and postoperative), MP retinal sensitivity (postoperative) and pre and postoperative visual acuity (logMAR)

		Visual acuity (logMAR)	
		Pre-op	Post-op
OCT full thickness Pre-op	Fovea	0.11 (0.49)	−0.26 (0.09)
	Average thickness	0.01 (0.94)	−0.11(0.49)
	Macular volume	0.02 (0.91)	−0.10 (0.50)
OCT full thickness Post-op	Fovea	0.42 (0.005)*	−0.02(0.91)
	Average thickness	0.02 (0.90)	−0.002 (0.99)
	Macular volume	0.02 (0.89)	−0.001 (0.99)
GCC thickness Pre-op	Average thickness	−0.09 (0.57)	−0.04 (0.79)
GCC thickness Post-op	Average thickness	−0.32 (0.03)*	0.08 (0.61)
MP Post-op	Fovea	−0.38 (0.01)*	−0.07 (0.64)
	Average sensitivity	−0.23 (0.13)	0.07 (0.64)

OCT: optical coherence tomography; GCC: ganglion cell complex. which corresponds to the macular retinal nerve fiber layer (RNFL) plus ganglion cell layer/inner plexiform; *: p < 0.05 values ​​obtained by Pearson correlation test. pre-op: preoperative. post-op: postoperative

We also assessed the correlation between MP retinal sensitivity and GCC and full-macular thickness measurements. A significant correlation was found between RS and full-macular thickness in the temporal and superior outer sectors after surgery (Table 6). However, no significant correlation was observed between preoperative OCT parameters and postoperative microperimetry RS values. A significant correlation was found between da GCC thickness and microperimetry RS at temporal inner, inferior inner, temporal outer, inferior outer sectors and for the average thickness (Table 6).

**Table 6:** Correlation between full-macular and ganglion cell complex thickness measured by preoperative and postoperative OCT and retinal sensitivity measured by postoperative microperimetry (MP) (9 sectors plus mean sensitivity)

Full-macular thickness	Retinal sensitivity (MP)	p-value	GCC thickness	Retinal sensitivity (MP)	p-value
Fovea			Fovea		
Pre-op	−0.20	0.19	Pre-op	−0.20	0.20
Post-op	−0.16	0.29	Post-op	−0.22	0.16
Temporal inner			Temporal inner		
Pre-op	0.04	0.77	Pre-op	0.08	0.59
Post-op	0.07	0.65	Post-op	0.37	0.01*
Superior inner			Superior inner		
Pre-op	−0.15	0.33	Pre-op	−0.16	0.31
Post-op	0.13	0.41	Post-op	−0.10	0.51
Nasal inner			Nasal inner		
Pre-op	−0.07	0.66	Pre-op	0.07	0.66
Post-op	0.08	0.62	Post-op	0.12	0.44
Inferior inner			Inferior inner		
Pre-op	−0.01	0.93	Pre-op	0.11	0.48
Post-op	0.05	0.73	Post-op	0.33	0.03*
Temporal outer			Temporal outer		
Pre-op	0.14	0.38	Pre-op	0.18	0.26
Post-op	0.33	0.03*	Post-op	0.33	0.03*
Superior outer			Superior outer		
Pre-op	0.15	0.34	Pre-op	0.29	0.06
Post-op	0.31	0.04*	Post-op	0.28	0.07
Nasal outer			Nasal outer		
Pre-op	0.10	0.54	Pre-op	0.16	0.30
Post-op	0.29	0.06	Post-op	0.12	0.44
Inferior outer			Inferior outer		
Pre-op	0.01	0.96	Pre-op	0.11	0.47
Post-op	0.14	0.38	Post-op	0.31	0.04*
Average thickness	Mean sensitivity		Average thickness	Mean sensitivity	
Pre-op	0.01	0.97	Pre-op	0.14	0.38
Post-op	0.22	0.16	Post-op	0.51	0.001*
Macular Volume	Mean sensitivity				
Pre-op	0.02	0.92			
Post-op	0.22	0.16			

OCT: optical coherence tomography; GCC: ganglion cell complex. which corresponds to the macular retinal nerve fiber layer (RNFL) plus ganglion cell layer/inner plexiform; *: p < 0.05 values ​​obtained by Pearson correlation test

## Discussion

The results of the present study revealed that all patients undergoing surgical removal of ERM associated with ILM peeling experienced significant improvement in VA 6 months post-surgery [3, 13, 14]. Consistent with findings from prior studies, pre-operative BCVA emerged as a crucial prognostic factor. In our study, patients with the highest pre-surgery VA exhibited better postoperative visual outcomes.

Another notable observation was the correlation between preoperative VA and both full-macular thickness and retinal sensitivity at the fovea postoperatively. This relationship likely reflects less anatomical and functional recovery in more advanced cases. Interestingly, our study found no correlation between preoperative macular thickness (both full-macular and GCC thickness) and postoperative visual acuity. Similarly, Yildiz et al. reported no correlation between central thickness of the fovea and final VA in multivariate analyses [15]. Conversely, Lee demonstrated that preoperative central foveal thickness correlates with visual improvement after ERM surgery [10]. Furthermore, our results indicated that retinal sensitivity (RS) values correlate with the full-macular and GCC thickness measurements after surgery. These findings differ from those reported by Pilli et al., which demonstrated a weak correlation between full-macular thickening and RS assessed by MP in ERM patients [16]. Our results show that the correlation between retinal sensitivity and thickness parameters was stronger with GCC thickness. This indicates that, as expected, assessing this layer more accurately reflects the degree of retinal sensitivity impairment compared to the total retinal thickness.

Although the presence of an epiretinal membrane can cause thickening and distortion across all layers of the retina, it primarily originates on the inner surface. Consequently, it is expected that the anatomical distortion, combined with the mechanical trauma from surgical peeling, can lead to structural changes and alterations in the thickness of the inner retina. Our findings indicate that GCC thickness was higher before surgery and remained thicker in the four inner sectors, as well as showing an overall higher average thickness 6 months post-surgery. However, GCC thickness was significantly reduced in the outer sectors. These findings suggest a correlation between mechanical trauma, anatomical distortion, and the thinning or atrophy of the ganglion cell/inner plexiform layer. The discrepancy in the pattern of GCC involvement between the inner and outer sectors is intriguing and can be partially attributed to the extent and intensity of ERM involvement. Before surgery, the GCC thickness is proportionally thicker in the inner sectors compared to the outer ones. After surgery, GCC thickness significantly reduces in all sectors. However, GCC atrophy may be more easily detected in the postoperative period in areas where retinal thickening was less intense.

While the thickening of GCC in ERM patients represents the contraction and distortion of this glial tissue proliferation of the surface of the retina, the thinning of this layer maybe due to ganglion cell damage and loss [10]. We believe that a combination of tangential traction caused by ERM, chronic inflammation, and mechanical trauma during the peeling of ERM and ILM may have contributed to the thinning of GCC thickness after surgery in these patients. [10]

In accordance with our findings, previous studies have shown that the GCL/IPL thickness are reduced after ERM peeling surgery. Park et al. retrospectively evaluated 58 eyes with idiopathic ERM that underwent to PPV to ERM peeling and found that GCC thickness was significantly lower than in control eyes, 6 months after surgery [8]. They also demonstrated that the reduction of GCC is associated with worse VA. In another retrospective study including 62 eyes with ERM, the authors demonstrated that macular GCL/IPL thickness was significantly lower 6 months after surgery [10]. In this study, the GCL/IPL reduction correlated with worse preoperative BCVA and lower macular visual field mean sensitivity values assessed by standard automated perimetry (Humphrey VF test).

It is important to emphasize that despite significant visual improvement 6 months after surgery, RS values were significantly worse in patients compared to controls. Our findings align with those of other studies, such as one recently published by Xu Z et al., which demonstrated that eyes with ERM had worse RS than control eyes, despite improvements in RS 6 months after surgery [17]. Reduced retinal sensitivity values may correlate with the persistent thickness changes found in the postoperative period of patients with ERM undergoing treatment, despite the subtle correlations demonstrated in our study. On the other hand, other structural aspects must be considered beyond just thickness measurements, as the presence of structural changes such as DRIL, outer retina changes, and the presence of intraretinal cysts may exert a greater influence on postoperative retinal sensitivity, as we recently demonstrated. [12]

Our study has limitations, including a relatively small sample size and the absence of preoperative microperimetry examinations. However, using normal control eyes was advantageous as it enabled the comparison of postoperative retinal sensitivity values, revealing that these values were reduced compared to those of normal control eyes.

## Conclusion

In summary, our results demonstrate that ERM peeling can improve visual acuity in the postoperative period. However, retinal sensitivity assessed by MP may not fully restore, remaining significantly lower when compared to normal controls. Both full-macular and GCC thickness measurements were reduced 6 months after surgery. However, significant thinning of the GCC thickness was observed, indicating the occurrence of some degree of GCL/IPL atrophy. Furthermore, our results have demonstrated correlations among various OCT thickness parameters, particularly for GCC thickness measurements. Our results suggest that ganglion cell layer integrity may play an important role in visual function after ERM surgery, and that MP may help better understand the correlations between structural and functional findings during the postoperative period following ERM peeling surgery.

## Data Availability

No datasets were generated or analysed during the current study.

## Abbreviations

GCC: Ganglion cell complex
MP: Microperimetry
ERM: Macular epiretinal membrane
RS: Retinal sensitivity
PPV: Pars-plana vitrectomy
ILM: Internal limiting membrane
BCVA: Best-corrected visual acuity
OCT: Optical coherence tomography
ETDRS: Early treatment diabetic retinopathy study
GCL/IPL: Ganglion cell/inner plexiform layer
VA: Visual acuity
DRIL: Disorganization of the retinal inner layers
IOL: Intra ocular lens
logMAR: Logarithm of the minimum angle of resolution
cpm: Cuts per minute
SS-OCT: Swept-source optical coherence tomography
3D: Tridimensional
RPE: Retinal pigment epithelium
mm: Milimetre
μm: Micrometre
IOP: Intraocular pressure
mmHg: Millimetre of mercury
ASB: Apostilb
dB: Decibel
CD/mm^2^: Candela per square metre
ANOVA: Analysis of variance
AROC: Area under receiver operating characteristic *curve*
